# A Prognostic Model for Colon Cancer Patients Based on Eight Signature Autophagy Genes

**DOI:** 10.3389/fcell.2020.602174

**Published:** 2020-11-26

**Authors:** Jiasheng Xu, Siqi Dai, Ying Yuan, Qian Xiao, Kefeng Ding

**Affiliations:** ^1^Department of Colorectal Surgery and Oncology, Key Laboratory of Cancer Prevention and Intervention, Ministry of Education, The Second Affiliated Hospital, Zhejiang University School of Medicine, Hangzhou, China; ^2^Zhejiang University Cancer Center, Hangzhou, China; ^3^Department of Medical Oncology, The Second Affiliated Hospital, Zhejiang University School of Medicine, Hangzhou, China

**Keywords:** colon cancer, autophagy genes, prognostic model, TCGA, prognostic markers

## Abstract

**Objective:**

To screen key autophagy genes in colon cancer and construct an autophagy gene model to predict the prognosis of patients with colon cancer.

**Methods:**

The colon cancer data from the TCGA were downloaded as the training set, data chip of GSE17536 as the validation set. The differential genes of the training set were obtained and were analyzed for enrichment and protein network. Acquire autophagy genes from Human Autophagy Database www.autophagy.lu/project.html. Autophagy genes in differentially expressed genes were extracted using R-packages limma. Using LASSO/Cox regression analysis combined with clinical information to construct the autophagy gene risk scoring model and divide the samples into high and low risk groups according to the risk value. The Nomogram assessment model was used to predict patient outcomes. CIBERSORT was used to calculate the infiltration of immune cells in the samples and study the relationship between high and low risk groups and immune checkpoints.

**Results:**

Nine hundred seventy-six differentially expressed genes were screened from training set, including five hundred sixty-eight up-regulated genes and four hundred eight down regulated genes. These differentially expressed genes were mainly involved: the regulation of membrane potential, neuroactive ligand-receptor interaction. We identified eight autophagy genes *CTSD*, *ULK3*, *CDKN2A*, *NRG1*, *ATG4B*, *ULK1*, *DAPK1*, and *SERPINA1* as key prognostic genes and constructed the model after extracting the differential autophagy genes in the training set. Survival analysis showed significant differences in sample survival time after grouping according to the model. Nomogram assessment showed that the model had high reliability for predicting the survival of patients with colon cancer in the 1, 3, 5 years. In the high-risk group, the infiltration degrees of nine types of immune cells are different and the samples can be well distinguished according to these nine types of immune cells. Immunological checkpoint correlation results showed that the expression levels of *CTLA4*, *IDO1*, *LAG3*, *PDL1*, *and TIGIT* increased in high-risk groups.

**Conclusion:**

The prognosis prediction model based on autophagy gene has a good evaluation effect on the prognosis of colon cancer patients. Eight key autophagy genes can be used as prognostic markers for colon cancer.

## Introduction

Colon cancer (carcinoma of colon) is a common malignant tumor in the gastrointestinal tract (Fearon and Vogelstein, 1990; Jemal et al., 2011). Its morbidity and mortality are only second to gastric cancer, esophageal cancer and primary liver cancer in malignant tumors of the digestive system (Meyerhardt and Mayer, 2005). Recent studies have shown that autophagy is involved in the occurrence and development of malignant tumors, neurodegenerative diseases, tissue fibrosis, cardiovascular diseases and immune diseases (Eskelinen, 2011). Depending on how the intracellular substrate is degraded and transported to the lysosomal cavity (Fulda and Kogel, 2015), autophagy can be divided into three types: macroautophagy, microautophagy, and chaperone-mediate autophgy.

Because of the prevalence of giant autophagy, in most cases giant autophagy is commonly referred to autophagy and is the most detailed form of autophagy being currently studied. Macroautophagy cannot only degrade macromolecules and organelles to protect cells, but also induce cell death mediated by autophagy, which is the main mechanism regulating the degradation of proteins and organelles in eukaryotic cells (Kondo et al., 2005; Maiuri et al., 2007; Martinet and De Meyer, 2009). In some tumors, autophagy can inhibit the growth of tumor cells and activate programmed cell death. In addition, autophagy can also regulate the occurrence and development of tumors through multiple mechanisms and signaling pathways, so that cells can survive under stress conditions. Therefore, the effects of autophagy on tumors are not unilateral or harmful, and their specific types of cancer should be differentiated (Mizushima, 2007; Cheng et al., 2013). Many key molecules related to autophagy have been extensively studied. It has been found that the process is highly conserved in yeast and human beings. A series of homologs of autophagy related genes in yeast have been widely found in mammals. Several core autophagy related factors play roles in two ubiquitination systems which are necessary for autophagy formation.

Although the autophagy response has been shown to be related to the occurrence and development of various tumors, the key genes affecting the prognosis of colon cancer patients in the autophagy response have yet to be confirmed. In this paper, we used machine learning methods to analyze the expression of 210 autophagy genes in 433 colon cancer patients and their prognostic value in colon cancer patients. In order to establish an accurate and reliable prognostic model for colon cancer patients, we constructed a prognostic model by using autophagy genes with significant prognostic relevance, and validated the model with external colon cancer datasets.

## Materials and Methods

### Ethics Approval Statement

No animals or humans were involved in this study. This study was carried out in accordance with the Declaration of Helsinki.

### Research Object

We downloaded the expression profile data and corresponding clinical information mRNA colon cancer (COAD) patients in The Cancer Genome Atlas (tcga)^1^ database, after excluding patients with incomplete information, 433 patients had complete survival information. In addition, we also downloaded the dataset numbered GSE17536 in the Gene Expression Omnibus (GEO database)^2^ database, the dataset contained 177 colon cancer patients, and 177 patients contained complete survival information. The samples were analyzed using Affymetrix Human Genome U133 Plus 2.0 Array platform to obtain data. Two hundred ten autophagy genes were collected in www.autophagy.lu/project.html, autophagy genes are detailed in Supplementary Table S1.

### Differential Gene Analysis

The differential expression gene analysis was based on the limma (PMID: 25605792) function package of R language (version3.5.2, the same below). The absolute values of differential expression multiples (Log2FC) of logarithmic transformation > 1 and FDR ≤ 0.05 were used as criteria to screen differentially expressed genes.

### Functional Enrichment Analysis

For the differentially expressed genes, we used the “clusterProfiler” (PMID: 22455463) function package in R to carry out the enrichment analysis of GO (including Biological Process, Molecular Function and Cellular Component) and KEGG Pathway enrichment analysis (DAVID^3^ online website can also be used to finish the same enrichment analysis). We thought the corresponding entries were significantly enriched at < 0.05.

### Protein–Protein Interaction (PPI) Networks

The STRING database is a database for analyzing and predicting protein functional connectivity and protein interactions. We used STRING (PMID: 30476243)^4^ to analyze the functional relationship and protein interaction of proteins, and used cytoHubba plug-in in Cytoscape (PMID: 14597658) (version 3.7.2) software to screen the key genes in PPI networks.

### LASSO Cox Regression Analysis

Based on the expression values of 210 autophagy genes, single factor Cox regression analysis was performed for colon cancer samples, autophagy genes significantly associated with colon cancer prognosis were screened with *P* < 0.05 as a threshold. Then LASSO Cox regression analysis with R package glmnet (PMID: 20808728) was used to further identify autophagy genes related to the prognosis of colon cancer, and the Risk Score of each sample was calculated using the screened autophagy genes through the following formula.

Risk score = $\sum_{i=1}^{n}{\text{Coef}}_{\text{i}}$ ^∗^ x_*i*_,

Coefi is the risk coefficient of each factor calculated by the LASSO-Cox model, Xi is the expression value of each factor and in this study, the expression value of mRNA is used. Then the optimal cutoff value of the Risk score was determined by R package survival, survminer, and bilateral test, patients were divided into Low Risk and High Risk groups according to the cutoff values.

### Survival Analysis

R language survival package and survminer package were used to estimate the overall survival rate of different groups based on the Kaplan-Meier method. R language survival ROC package (PMID: 10877287) plot time dependent subject work characteristics (ROC) curves. The multivariate Cox regression model was used to analyze whether Risk Score could independently predict the survival of patients with colon cancer independently of other factors.

### The Proportion of Immune Cell Infiltration and the Calculation of Tumor Purity

We used software CIBERSORT (PMID: 25822800) to calculate the relative proportions of 22 immune cells in each cancer sample. CICERSORT software is based on the gene expression matrix. CICERSORT software can use the deconvolution algorithm to characterize the composition of immune infiltrating cells using the preset 547 barcode genes based on gene expression matrix. The proportion of all estimated immune cell types in each sample was equal to 1. Using the R language estimate function package (PMID: 24113773) to calculate the tumor purity of each cancer sample.

### Establishment of Nomogram Prognosis Prediction Model

Nomogram is widely used to predict the prognosis of cancer. In order to predict the survival probability of patients in 1, 3, and 5 years, to predict patient survival probability for 1, 3, and 5 years, we established nomogram, and plotted nomogram calibration curves based on all independent prognostic factors determined by multivariate Cox regression analysis using the R language rms package to observe the relationship between the predicted probability and the actual incidence.

### Expression Verification of Prognosis-Related Genes

Verification of gene expression of the selected autophagy genes related to prognosis.

The Human Protein Atlas (HPA)^5^ was used to validate the expression of autophagy genes related to prognosis in colon cancer tumor tissues and normal tissues, and to compare whether the expression differences were consistent with the results of previous analysis.

### Statistical Analysis

Kaplan-Meier method was used to estimate the overall survival rate of different groups, and log-rank was used to test the significance of the difference between different groups. The difference of infiltration of immune cells in different groups was compared by using Wilcoxon signed rank sum test, and *P* < 0.05 was used as a significant threshold. Statistical analysis was made using R software, with version number v3.5.2.

### Ethical Approval and Consent to Participate

No animals or humans were involved in this study. This study was carried out in accordance with the Declaration of Helsinki.

## Results

### Analysis of Differentially Expressed Genes

**FIGURE 1:**
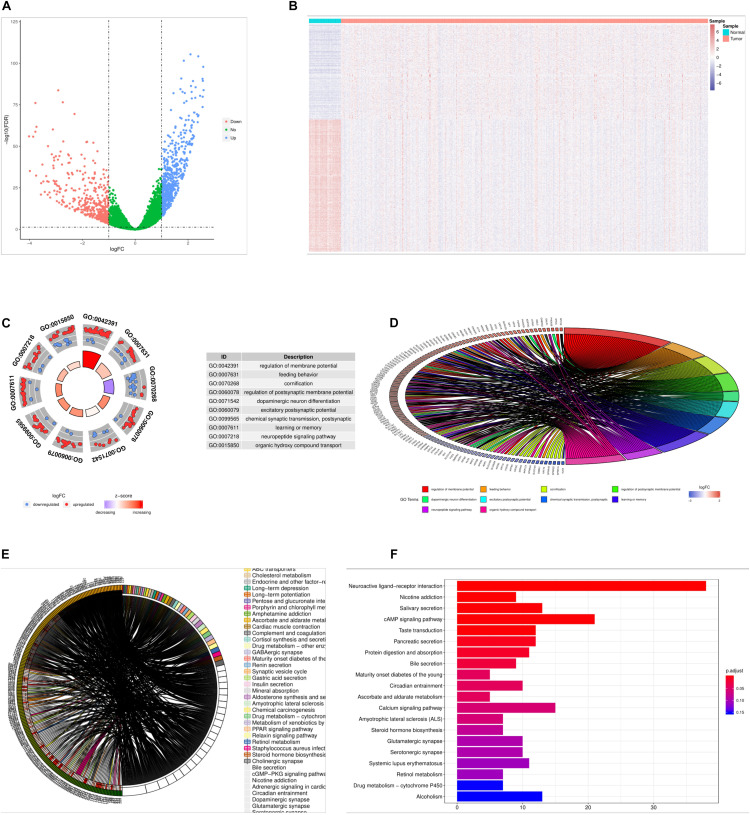
Results of differential gene analysis. **(A)** volcano plot of differentially expressed genes, the horizontal axis is the differential expression multiple (Log2FC), the longitudinal axis is—log10(fdr), the blue point is the up-regulated gene, and the red point is the down-regulated gene. **(B)** The heat map of the differentially expressed genes, the horizontal axis is the sample, the longitudinal axis is the different genes, the red indicates the high expression of the gene, and the blue indicates the low expression of the gene. **(C)** Circle graph of the top 10 GO terms with the most enriched genes. **(D)** Enriched the first 10 GO term and chord graphs with the largest number of genes, the right semicircle represented 10 GO term, the left semicircle represented the genes enriched in these 10 GO term. **(E)** Chord diagram of the main KEGG signaling pathway for gene enrichment. **(F)** Top 20 KEGG pathway, with the largest number of enriched genes, the horizontal axis of the map indicated the number of genes enriched, and the longitudinal axis indicated the names of each species.

In the TCGA dataset, we obtained 976 differentially expressed genes in cancer samples relative to the samples from the cancer samples, including 568 up-regulated genes and 408 down regulated genes (Figure 1A). The difference in the expression of differentially expressed genes between cancer and paracancerous samples was more obvious (Figure 1B).

### Go and KEGG Enrichment Analysis Results

Through GO and KEGG enrichment analysis, we found that 976 differentially expressed genes were significantly enriched in GO term, such as regulation of membrane potential, and the top 10 most significant differentially expressed genes GO terms were shown in Figures 1C,D, detailed results of the GO enrichment analysis were shown in Supplementary Table S1. The 976 differentially expressed genes were significantly enriched in Neuroactive ligand-receptor interaction and other KEGG Pathway, among the most significant of the first 20 pathways were shown in the detailed results of the Figures 1E,F, KEGG enrichment analysis in Supplementary Table S2.

### PPI Network Construction and Screening Results of Key Genes

**FIGURE 2:**
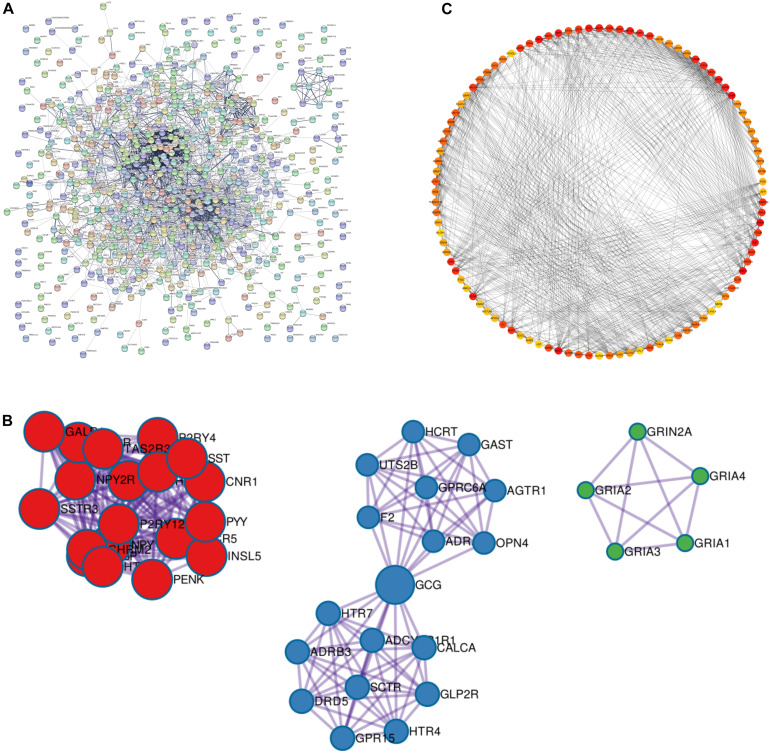
PPI network build results. **(A)** PPI network diagram, every dot in the network represents a node, the more lines connected to the dot, the larger the degree representing this node. It means that the gene on this node may be more important in the network structure. **(B)** The three main clustering modules in the PPI network. **(C)** MCC degree higher top 100 genes in the network screened by the algorithm, the darker the red color indicates the higher the degree.

We used STRING database to construct PPI networks for 976 differentially expressed genes, a threshold of minimum required interaction score > 0.4 was used to screen interaction pairs, PPI network had 634 nodes and 2,999 sides in Figure 2A. A node represented a gene, and the edges represent the interrelationships between them. Then we used Cytoscape software to analyze the whole PPI network. MCC algorithm was used to score each node in the network, and the top 100 genes were selected from large to small. The 100 genes were shown in detail in Supplementary Table S3. The deeper the color is, the higher the importance of nodes is (Figures 2B,C).

### Construction and Validation of a Prognostic Model

**FIGURE 3:**
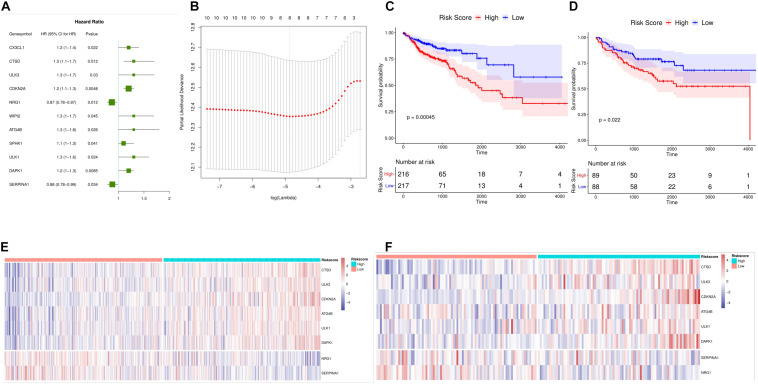
Establishment of a prognostic model for colon cancer. **(A)** Forest maps of 15 autophagy genes significantly associated with colon cancer prognosis. HR Hazard ratio, 95%CI 95% confidence interval. **(B)** LASSO Figure in the regression model to determine the tuning parameter lambda. the horizontal axis is log (lambda), and the longitudinal axis is the partial likelihood deviation value (partial likelihood Deviance). the corresponding Lambda value at the minimum of this value is the best, that is, the best Lambda value after taking the Log below the dotted line, and the number of variables corresponding above. **(C)** Survival curve Kaplan Meier TCGA data set, the horizontal axis is time, the longitudinal axis is survival, and different colors represent different groups. *P*-values are based on log-rank tests. **(D)** Kaplan Meier survival curve and time dependent ROC curve of (GEO) datasets. **(E,F)** Heat maps of mRNA expression of eight selected genes in high and low risk score samples of TCGA and GEO datasets. The horizontal axis is the sample, the longitudinal axis is the gene, the red represents the high expression, the blue represents the low expression, and the heat map shows the category of the sample with different colors above.

Using TCGA data sets, 210 autophagy gene expression values were used as continuous variables to conduct univariate Cox regression analysis, and Hazard ratio (HR) of each gene was calculated. *P* < 0.05 was selected as the threshold for screening. Finally, 11 genes were obtained, and the HR value of two genes was less than 1. There were nine genes with a HR value greater than 1 which were risk genes that were unfavorable to prognosis (Figure 3A). After that, we screened the 11 autophagy genes by LASSO Cox regression analysis. The optimal number of genes was determined to be 8 (minimum Figure 3B, lambda value) according to the lambda values corresponding to the number of different genes in the LASSO Cox analysis. The eight genes were *CTSD*, *ULK3*, *CDKN2A*, *NRG1*, *ATG4B*, *ULK1*, *DAPK1*, and *SERPINA1*.

Based on the expression of each gene and the regression coefficient of LASSO Cox regression analysis, a risk score model for predicting the survival of patients was established. Risk Score = (Express Value of *CTSD*^∗^0.08810216)+(Express Value of *ULK3*^∗^0.06755919)+(Express Value of *CDKN2A*^∗^0.08355253)+(Express Value of *NRG1*^∗^-0.11920988)+(Express Value of *ATG4B*^∗^0.07071560)+(Express Value of *ULK1*^∗^0.01917531)+ (Express Value of *DAPK1*^∗^0.12813474)+ (Express Value of *SERPINA1*^∗^-0.15361284). We calculated the risk score for each patient and divided the samples of TCGA dataset and GEO validation set into high-risk and low-risk groups according to the median. Survival analysis revealed that in TCGA and GEO datasets, High risk colon cancer samples showed poorer overall survival (Figures 3C,D) than those with low risk. At the same time, we found that there was a significant difference in the expression of the eight genes between the high-risk groups (Figures 3E,F). Overall, the results showed that the risk score (Risk Score) calculated using the evaluation model constructed by *CTSD, ULK3, CDKN2A, NRG1, ATG4B, ULK1, DAPK1*, and *SERPINA1* could better predict the prognosis of colon cancer patients.

### Risk Score Is an Independent Prognostic Marker for Colon Cancer

**FIGURE 4:**
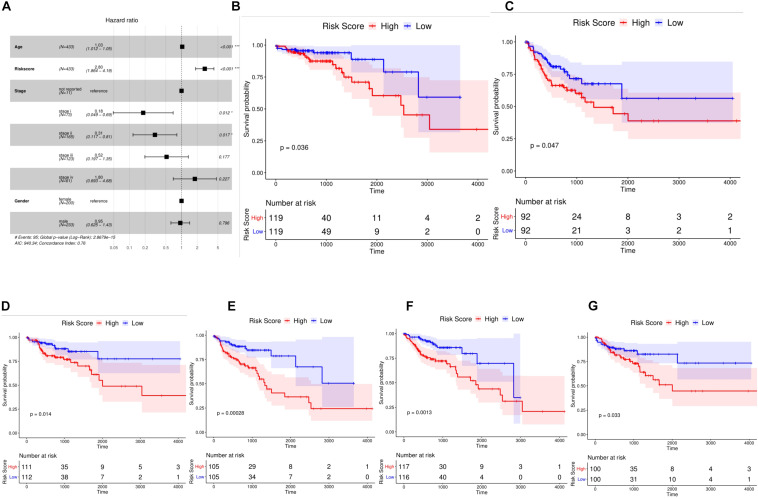
Risk score was independent prognostic marker for colon cancer. **(A)** Multivariate Cox regression analysis of forest plots. Compared with reference samples, samples with Hazard ratio greater than 1 had higher risk of death. Samples with Hazard ratio less than 1 had a lower risk of death. **(B,C)** Kaplan Meier survival curves of colon cancer samples with different Stage stages. **(D,E)** Kaplan Meier survival curves of colon cancer samples from different age groups. **(F,G)** Kaplan survival curves of colon cancer samples from different sexes.

We included four factors including age, TNMStage, gender, and Risk Score to conduct a multivariate Cox regression analysis to determine whether the risk score was an independent prognostic indicator. Results were shown in Figure 4A. Risk Score, TNMStage, and age were found to remain significantly associated with overall survival, with a higher risk of death in samples with a high risk score, which was a poor prognostic factor (*HR* = 2.8, 95% CI :1.864-4.19, *P* < 0.001).

To further explore the prognostic value of Risk Score in colon cancer specimens with different clinicopathological factors, including age, TNM Stage and sex, we regrouped the patients with colon cancer according to these factors and analyzed the survival of Kaplan-Meier. Stage I, Stage II, Stage III, and Stage IV samples were found (Figures 4B,C); ≤68 and 68 (Figures 4D,E); male and female samples (Figures 4F,G); The overall survival rates of the high-risk group were significantly lower than those of the low risk group. These results indicated that Risk Score could be used as an independent indicator to predict the prognosis of patients with colon cancer.

### The Nomogram Model Can Better Predict the Prognosis and Survival of Patients

**FIGURE 5:**
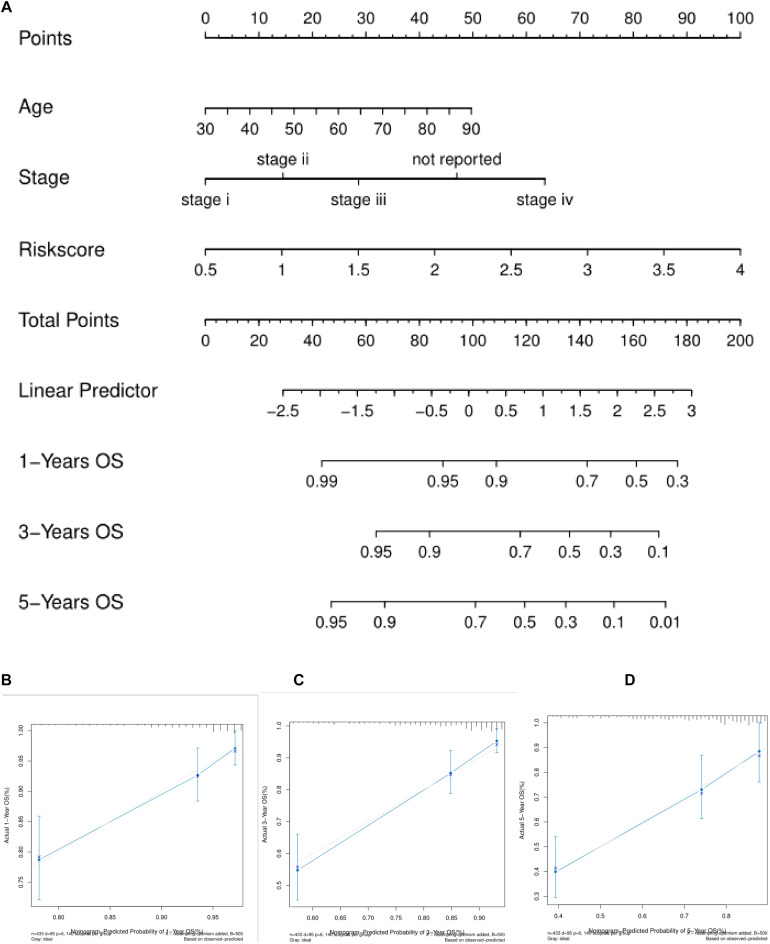
Nomogram predicts the survival of patients with colon cancer. **(A)** Nomogram to predict the probability of 1, 3, and 5 years OS in colon cancer patients. **(B–D)** Normative curve for predicting the probability of 1, 3, and 5 years OS colon cancer patients. the X axis represents nomogram predicted survival and the Y axis represents the actual survival.

We used the three independent prognostic factors of age, TNMStage and Risk Score to construct the nomogram model (Figure 5A). For each patient, three lines were drawn up to determine the Points. The sum of these Points was located on the “Total Points” axis, and then drew a line down from the Total Points axis to determine the probability that colon cancer patients will survive for 1, 3, and 5 years. The corrected curve in the calibration map was closer to the ideal curve (45 degree line with a slope of 1 at the origin of the coordinate axis) which indicated that the prediction was in good agreement with the actual results (Figures 5B–D).

### Immune Status of Colorectal Cancer Patients With High and Low Risk Group

**FIGURE 6:**
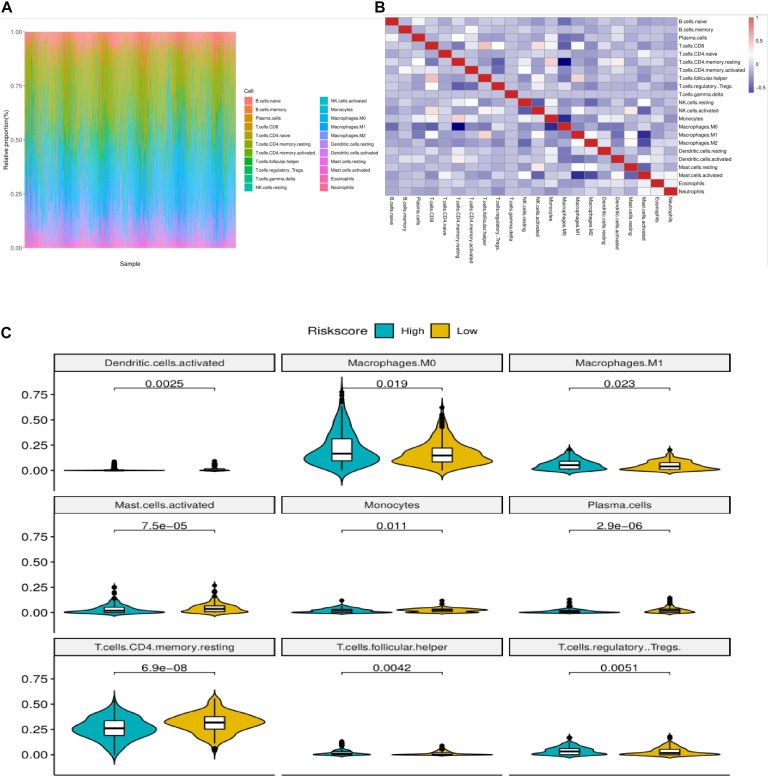
Correlation analysis of immune infiltration in colon cancer patients with high and low risk groups. **(A)** Relative proportion of immune infiltrating cells in all patients. **(B)** Correlation matrix of 22 immune cell proportions. Red represents positive correlation. Blue is negative correlation. The deeper the color is, the greater the correlation. **(C)** The violin diagram of immune cells with significant difference in high and low risk group, the horizontal axis is high and low risk group, the longitudinal axis is the relative infiltration ratio of immune cells, and the *p*-value is calculated by wilcoxn method.

**FIGURE 7:**
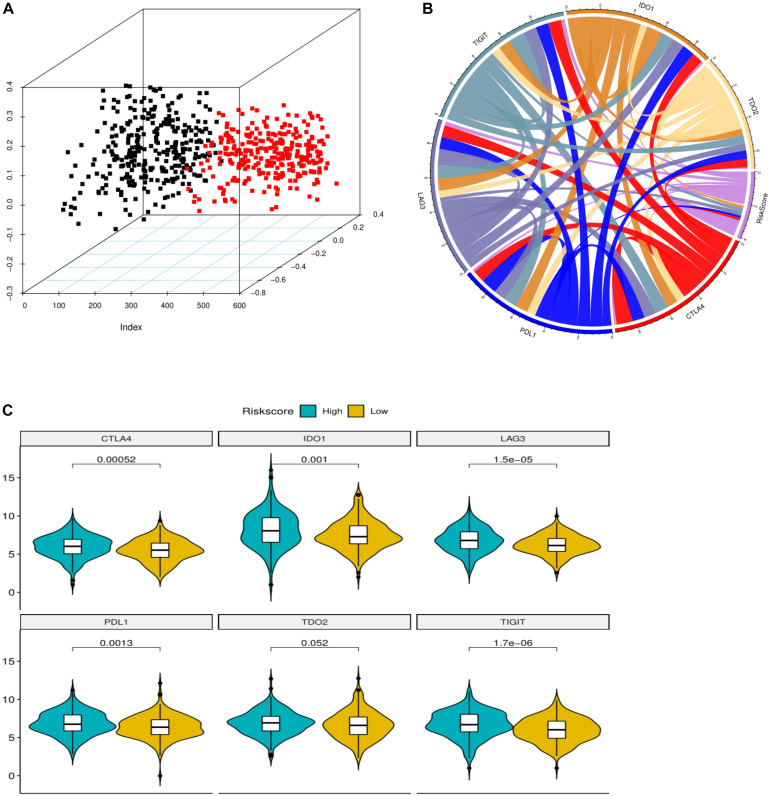
**(A)** PCA three dimensional clustering diagram. Different color points represented different types of samples. **(B)** Correlation chord maps (Chord diagram) of risk scores with six prominent immune checkpoint expression, the more connections between them represent the stronger correlation between them. **(C)** The immune checkpoint violin diagram with significant difference in expression in high and low risk groups. Different colors represent high and low risk groups. The longitudinal axis is the expression amount. The *p*-value is calculated by wilcoxon method.

We used CIBERSORT method combined with LM22 feature matrix to estimate the immune infiltration differences between 22 immune cells in colorectal cancer patients with high and low risk groups. Figure 6A summarized the results of immune cell infiltration in 433 colon cancer patients. The changes in the proportion of tumor infiltrating immune cells in different patients may represent the intrinsic characteristics of individual differences. The correlation between the infiltration ratios of different types of immune cells is relatively weak (Figure 6B). There was a significant difference in the proportion of nine kinds of immune cells in Macrophages between the high risk group and the low risk group (Figure 6C). The PCA analysis showed that the samples could be divided into high-risk group and low-risk group (Figure 7A) according to the clustering of these nine different immune cells, indicating that the content difference of immune cells may be the potential cause of the risk of sample height and height.

### Relationship Between Riskscore and Immunological Checkpoint Genes

The expression of immune checkpoints has become a biomarker for colon cancer patients to choose immunotherapy. We analyzed the correlation between patient risk score and key immune checkpoints (CTLA4, PDL1, LAG3, TIGIT, IDO1, TDO2), and found that the risk score was correlated with them (Figure 7B). Moreover, the six immunoassay checkpoints were in addition to TDO2, the other five immunocheck points had significant difference in the expression of high risk colon cancer patients (Figure 7C), and the expression level of high risk colon cancer group was significantly higher than that of low risk colon cancer group (*P* < 0.05).

### Immunohistochemical Verification of Prognostic Genes

**FIGURE 8:**
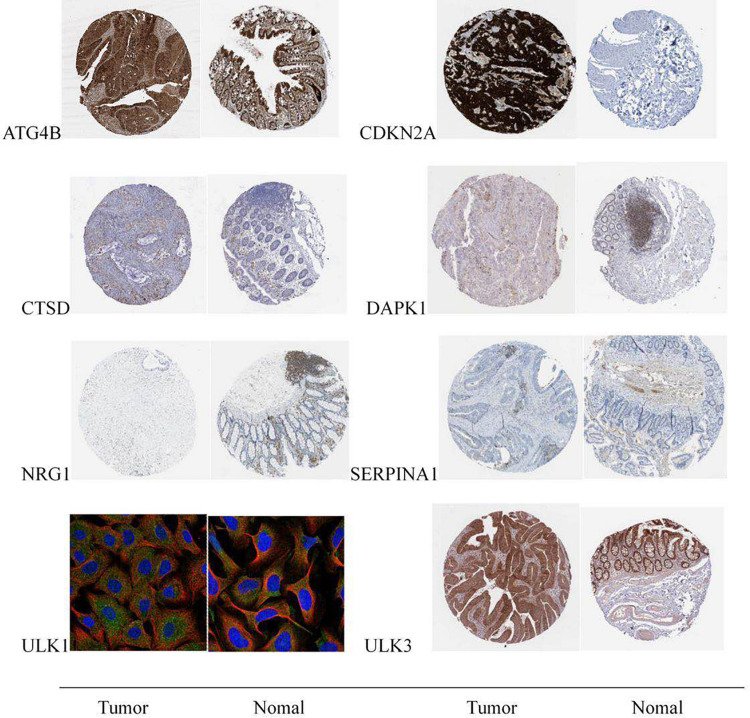
Expression of eight prognostic-related autophagy genes in colon cancer tumor tissues and normal tissues.

The data verification results of the HPA database indicated that the expression of ULK1 in cancer and adjacent tissues had not been detected in the database, and the expression of the remaining seven genes in cancer and adjacent tissues could be verified. Among them, NRG1 gene was not significantly expressed in tumor and normal tissues, and there was no significant difference in expression. Compared with normal tissues, the expressions of *CTSD, ULK3, CDKN2A, ATG4B, and DAPK1* in tumor tissues were significantly up-regulated, and the expression of *SERPINA1* in tumor tissues was significantly down-regulated; the verification results were basically consistent with the research analysis results (Figure 8).

## Discussion

Colon cancer is one of the main malignant tumors of the gastrointestinal tract, around 600 thousand people die of colon cancer every year (American Cancer Society, 2017; Siegel et al., 2017; Bray et al., 2018). With the improvement of surgical method and follow-up treatment, the 5 year survival rate of colon cancer in developed countries is close to 65% (Miller et al., 2016). However, for patients with cancer penetrating the intestinal wall or distant metastasis, their mortality is very high (Misale et al., 2012; Edwards et al., 2014; Fang et al., 2017). Therefore, it is urgent to find some new therapeutic targets which are closed to the prognosis of the patient.

In recent years, autophagy has been found to be closely related to the occurrence and development of tumors and the prognosis of cancer patients (Kroemer et al., 2010; Mizushima and Komatsu, 2011). Autophagy is very important for physiological processes such as cell development, differentiation, tissue remodeling and so on, and is very important for maintaining cell homeostasis (Kroemer and Levine, 2008; Li et al., 2010; Bhardwaj et al., 2018). Current studies have indicated that ABHD5, PFKFB3, oxaliplatin, for example, can play an antitumor role by regulating autophagy. However, a comprehensive study of the correlation between autophagy defects and metabolic dysfunction in colon cancer and its close relationship as well as functional interdependence in tumorigenesis have not been conducted (Kumar et al., 2012; Yan et al., 2014). At present, there are no studies that specifically analyze which genes in autophagy genes have an impact on the prognosis of colon cancer patients and what are the related biological response processes. It is of great significance to find which autophagy genes that play an important role in the development of the colon cancer and the prognosis of the patient. We used machine learning methods to analyze the data of a large number of colon cancer patients, constructed a prognostic evaluation model of colon cancer patients based on autophagy genes and verified the efficiency of the model using external data sets; using immunohistochemistry to verify the prognosis-related autophagy genes. In this study, we used colon cancer samples from TCGA as training group, GSE17536 as validation group, eight key prognostic autophagy genes in colon cancer were screened and identified, they were modeled by differential analysis, PPI network construction, COX single factor analysis, and LASSO Cox regression analysis. We used machine learning methods to analyze the data of a large number of colon cancer patients, constructed the prognostic evaluation model of colon cancer patients based on autophagy genes and verified the efficiency of the model using external data sets. Immunohistochemistry was used to verify the prognosis-related autophagy genes. Our results suggested that the constructed model can well distinguish colon cancer patients and predict prognosis, thereby helping to develop individualized treatment options based on patients’ risk.

We identified a group of ARGs that predict the prognosis of colon cancer patients. Most of these genes have been reported in previous studies to be closely related to the prognosis of colon cancer or other malignancies. Lu et al. (2017); Shen et al. (2017), and Yan et al. (2019) reported that *CTSD* promotes the proliferation, migration, invasion and metastasis of hepatocellular carcinoma cells. Goruppi et al. (2017) reported that *ULK3* links two main signaling pathways involved in cancer-associated fibroblasts conversion of several tumor types and is an attractive target for stroma-focused anti-cancer intervention. *CDKN2A* inhibition combined with TAE therapy can promote tumor cell necrosis in hepatocellular carcinoma rats (Gade et al., 2017). The phosphorylation of *ATG4B* at Ser34 promoted the Warburg effect and the decrease of oxygen consumption in hepatocellular carcinoma cells (Ni et al., 2018). It can also alleviate intestinal inflammatory reaction and intestinal epithelial apoptosis through autophagy pathway (Li et al., 2018), while *ULK1* is the key gene of autophagy. Liu et al. (2019) reported that blocking *AMPK/ULK1*-dependent autophagy promoted apoptosis and initiated autophagy simultaneously, and suppressed colon cancer growth. The increased expression of *DAPK1* in cholangiocarcinoma promotes the apoptosis of cholangiocarcinoma cells and alleviates the autophagy induced by cholangiocarcinoma cells (Thongchot et al., 2018). The constructed model can well distinguish colon cancer patients and predict prognosis, thereby helping to develop individualized treatment options based on patient risk.

The aim of this study is to construct a model composed of prognostic autophagy genes which can well distinguish colon cancer patients and predict prognosis. The model we constructed included *CTSD, ULK3, CDKN2A, NRG1, ATG4B, ULK1, DAPK1*, and *SERPINA1* these eight genes. Among them, *CTSD, ULK3, CDKN2A, ATG4B, ULK1, DAPK1* are beneficial genes that are benefit to prognosis. *NRG1* and *SERPINA1* are dangerous genes that are not conducive to prognosis (Figure 3A). We performed a multivariate Cox regression analysis and Risk Score, the results showed that the survival time of the high-risk group was significantly lower than that of the low risk group (Figures 4D–G). This shows that our model can be used as an independent prognostic factor for colon cancer patients. According to the nomogram model, the survival rate of colon cancer patients is consistent with the actual situation. This indicates that the constructed model can well distinguish colon cancer patients and predict prognosis. The results of immune cell infiltration in colon cancer samples showed that there was a significant difference in the infiltration ratio and other nine immune cells in high and low risk groups (Figure 6C). PCA results showed that the samples can be well differentiated according to these nine immune cells (Figure 7A). This indicates that the autophagy gene may affect the tumor cells by affecting the immune cells. The immune checkpoint correlation study of the samples grouped according to the model found that the expression of *CTLA4*, *PDL1*, *LAG3*, *TIGIT*, *IDO1* in the high risk group was significantly higher than that in the low risk group (Figure 7C). It is suggested that the poor prognosis of patients with high risk colon cancer may be due to immunosuppressive microenvironment. According to our research, the models constructed from these eight autophagy genes can well predict the prognosis of patients with colon cancer. We think these eight genes are biomarkers for predicting the prognosis of colon cancer patients, and may become new research targets for colon cancer patients. Our research identified the autophagy genes associated with prognosis and provided a new method for evaluating the prognosis of colon cancer patients. However, there are still some limitations in our study. The prognostic model still needs to be further validated in other independent large sample cohorts to ensure the reliability of our model. Functional experiments are needed to further reveal the possible mechanisms for predicting the role of autophagy genes.

## Conclusion

We constructed an autophagy gene model closely related to the prognosis of colon cancer patients by analyzing the samples from patients with colon cancer. The model contains eight autophagy genes, including *CTSD*, *ULK3*, *CDKN2A*, *NRG1*, *ATG4B*, *ULK1*, *DAPK1*, and *SERPINA1*. These eight genes are closely related to the autophagy process of tumor development and development. We think that the models constructed from these eight genes can predict the prognosis of colon cancer patients well. And these eight genes may become biological targets regulating cell autophagy and treating colon cancer patients.

## Data Availability Statement

The datasets generated for this study can be found in the online repositories. The names of the repository/repositories and accession number(s) can be found in the article/Supplementary Material.

## Author Contributions

JX: research design and drafting the manuscript. SD: experiment implementation. YY: literature search and experiment implementation. QX: help modify articles and collate references. KD: review and revision of the manuscript and writing guidance. All authors contributed to the article and approved the submitted version.

## Conflict of Interest

The authors declare that the research was conducted in the absence of any commercial or financial relationships that could be construed as a potential conflict of interest.

## Footnotes

1 https://tcga-data.nci.nih.gov/tcga/

2 https://www.ncbi.nlm.nih.gov/geo/

3 https://david.ncifcrf.gov/

4 https://string-db.org/,version 11.0

5 http://www.proteinatlas.org/

## Abbreviations

COAD: Colon cancer
RMA: Multi-Array Average
PPI: Protein–protein interaction
MCC: Maximum neighborhood component
ROC: Receiver operating characteristic
HPA: Human Protein Atlas
HR: Hazard ratio.
